# Supplementary material for: Transport pathways and kinetics of cerebrospinal fluid tracers in mouse brain observed by dynamic contrast-enhanced MRI
Source: Sci Rep. 2023 Aug 24;13:13882. doi: 10.1038/s41598-023-40896-x (PMC10449788; doi:10.1038/s41598-023-40896-x)
Supplement: Supplementary file 1 — Supplementary Information. [file 41598_2023_40896_MOESM1_ESM.pdf]

Scientific Reports

Transport Pathways and Kinetics of Cerebrospinal Fluid Tracers in Mouse Brain Observed by Dynamic Contrast-Enhanced MRI

Yuran Zhu[1], Guanhua Wang[2], Chaitanya Kolluru[1], Yuning Gu[1], Huiyun Gao[3,4], Jing Zhang[5], Yunmei Wang[3,4], David L. Wilson[1,6], Xiaofeng Zhu[5], Chris A. Flask[1,6,7], Xin Yu[1,6,8]

[1]Department of Biomedical Engineering, Case Western Reserve University, Cleveland, Ohio, USA
[2]Department of Biomedical Engineering, University of Michigan, Ann Arbor, Michigan, USA
[3]Cardiovascular Research Institute, Case Western Reserve University, Cleveland, Ohio, USA
[4]Department of Medicine, Case Western Reserve University, Cleveland, Ohio, USA
[5]Department of Biostatistics, Population and Quantitative Health Sciences, Case Western Reserve University, Cleveland, Ohio, USA
[6]Department of Radiology, Case Western Reserve University, Cleveland, Ohio, USA
[7]Department of Pediatrics, Case Western Reserve University, Cleveland, Ohio, USA
[8]Department of Physiology and Biophysics, Case Western Reserve University, Cleveland, Ohio, USA

Running headline: **Transport of CSF tracers in mouse brain**

Address correspondence to:

Xin Yu, Sc.D.,
Wickenden 430
10900 Euclid Avenue
Cleveland, OH 44106, USA
Tel: 216-368-3918
Email: xin.yu@case.edu

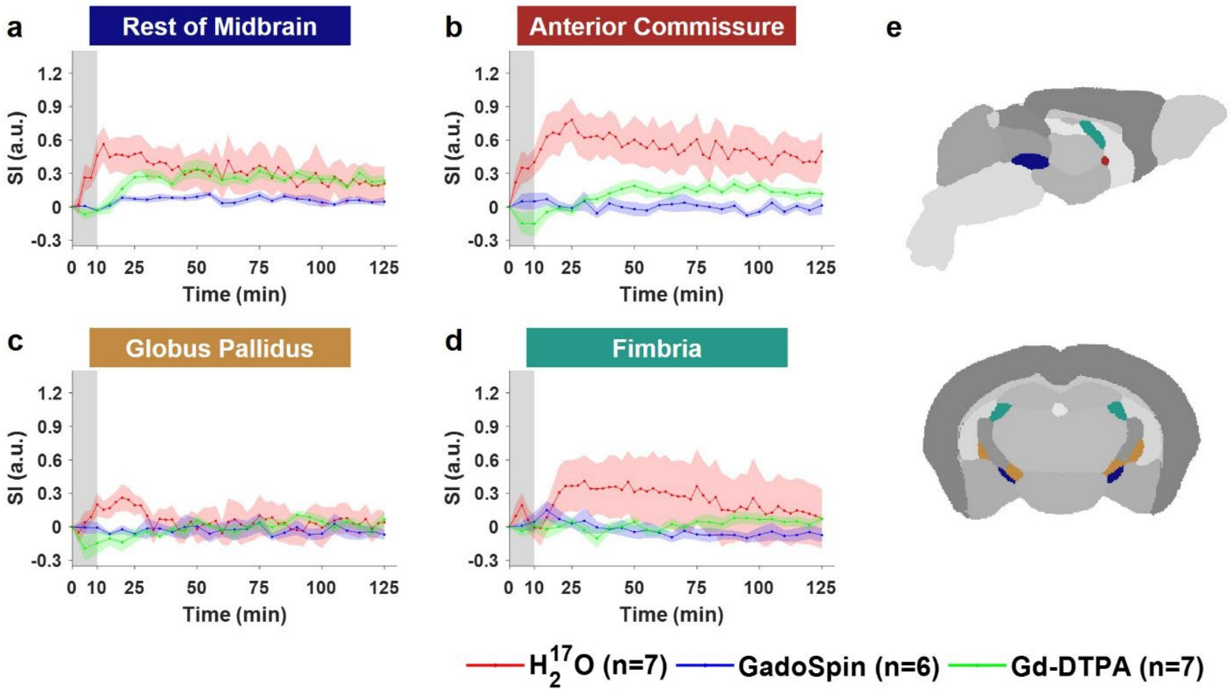

Fig. S1 Contrast agent transport in the rest of midbrain, anterior commissure, globus pallidus, and fimbria. **a-d:** Time courses of signal changes in the selected ROIs. Gray bands indicate the period of contrast agent infusion. Red, blue, and green lines represent the mean time courses of signal changes induced by $H_2{}^{17}O$, GadoSpin, and Gd-DTPA, respectively. Shaded areas represent standard errors. **e:** Segmentation of selected ROIs.

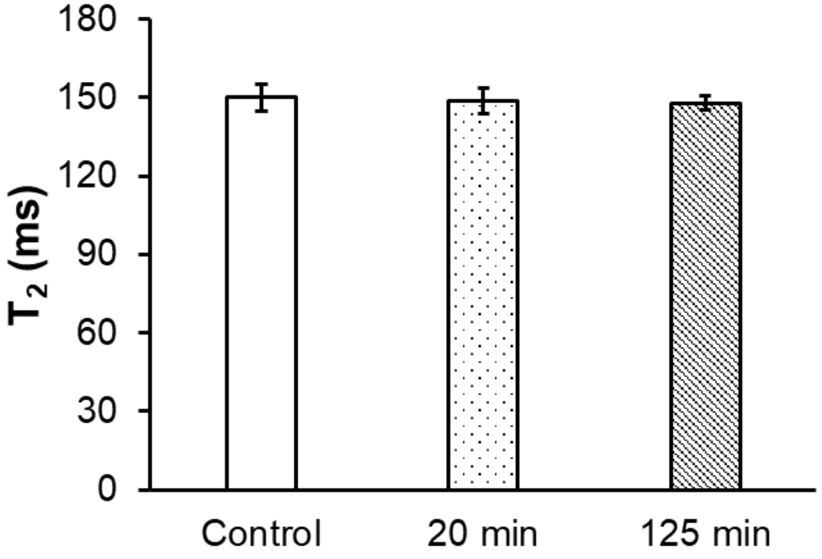

Fig. S2 T_2 of plasma from mice without $H_2{}^{17}O$ infusion (control) and at 20 and 125 min after $H_2{}^{17}O$ infusion.

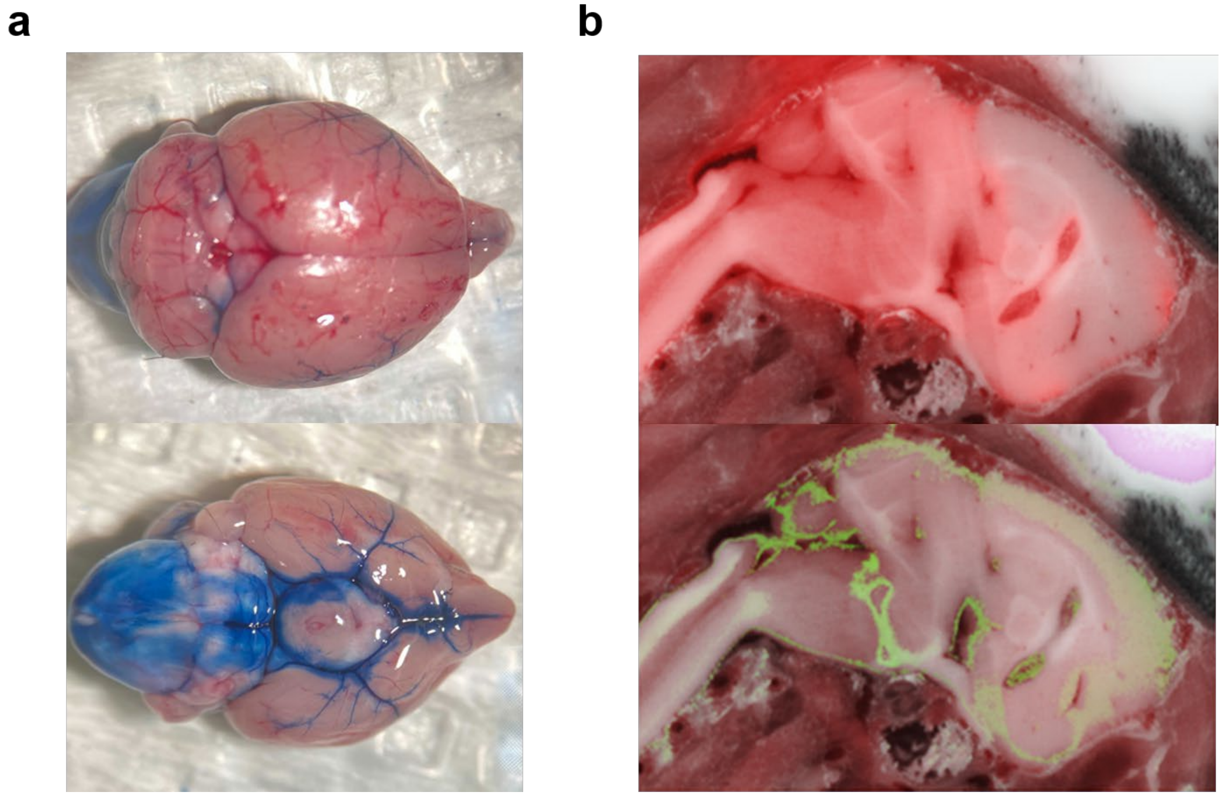

Fig. S3 Validation of intracisternal tracer delivery. **a:** Bright-field microscopy of Evans blue staining. Top: dorsal view; bottom: ventral view. **b:** Cryoimaging of co-injected CF594 hydrazide (MW=740 Da, red, top) and FITC-dextran (MW=2,000 kDa, green, bottom). CF594 hydrazide showed penetration into the parenchyma while Evans blue and FITC-dextran were confined to the subarachnoid and perivascular spaces.

Table. S1 Volumes of each region-of-interest.

Structure Name	Volume (mm^3)
Whole Brain	493.2
Neocortex	165.4
Brain Stem	73.6
Cerebellum	63.5
Olfactory Bulbs	31.1
Thalamus	25.8
Caudate Putamen	24.5
Hippocampus	24.4
Basal Forebrain	13.3
Rest of Midbrain	12.9
Amygdala	12.4
Hypothalamus	12.3
CC & EC	8.6
Superior Colliculi	8.2
Inferior Colliculi	5.2
Central Gray	3.9
Globus Pallidus	2.6
Internal Capsule	2.0
Fimbria	2.0
Lateral Ventricles	1.0
Anterior Commissure	0.6
